# Metformin Treatment in PCOS Pregnancies Reduces Maternal Infections and Increases the Risk of Allergies and Eczema in the Offspring: Post Hoc Analyses of Two Randomised Controlled Trials and One Follow‐Up Study

**DOI:** 10.1111/1471-0528.18320

**Published:** 2025-08-11

**Authors:** Mariell Ryssdal, Johanne E. Skage, Anders H. Jarmund, Liv Guro E. Hanem, Tone S. Løvvik, Guro F. Giskeødegård, Ann‐Charlotte Iversen, Eszter Vanky

**Affiliations:** ^1^ Department of Clinical and Molecular Medicine Norwegian University of Science and Technology Trondheim Norway; ^2^ Childrens Clinic, St. Olavs Hospital Trondheim University Hospital Trondheim Norway; ^3^ Department of Obstetrics and Gynecology, St. Olavs Hospital Trondheim University Hospital Trondheim Norway; ^4^ HUNT Center for Molecular and Clinical Epidemiology, Department of Public Health and Nursing Norwegian University of Science and Technology Trondheim Norway

**Keywords:** allergy, asthma, eczema, infections, metformin, polycystic ovary syndrome, pregnancy

## Abstract

**Objective:**

To evaluate the effect of metformin on immunological outcomes in pregnant women with polycystic ovary syndrome (PCOS) and their offspring.

**Design:**

Post hoc analyses of two randomised controlled trials (PregMet and PregMet2) and one follow‐up study (PedMet).

**Setting:**

Women followed at multiple hospitals in Norway, Sweden and Iceland, and offspring followed at multiple hospitals in Norway.

**Population or Sample:**

Pregnant women with PCOS, randomised to metformin or placebo from the first trimester to delivery, and offspring exposed to metformin or placebo in utero.

**Methods:**

Maternal infections and allergic diseases in offspring were compared using logistic regression. Maternal body mass index (BMI), offspring BMI *z*‐score and maternal infections were evaluated as effect modifiers or mediators.

**Main Outcome Measures:**

Incidence of maternal infections during pregnancy, delivery, and postpartum, and allergic diseases in offspring at 8‐year follow‐up.

**Results:**

Altogether 634 women and 145 offspring were included. Women treated with metformin experienced fewer overall infections during pregnancy (OR = 0.68, 95% CI: 0.50–0.93), particularly viral infections (OR = 0.71, 95% CI: 0.51–0.99). Offspring exposed to metformin in utero had a higher incidence of allergies (OR = 4.83, 95% CI: 1.47–21.8) and eczema (OR = 2.42, 95% CI: 1.14–5.33). Maternal BMI did not modify the effect of metformin, and offspring BMI *z*‐score or maternal infections did not mediate the relationship between metformin treatment and increased allergies and eczema in offspring.

**Conclusions:**

Metformin treatment in pregnant women with PCOS reduced maternal infections during pregnancy and increased the incidence of allergies and eczema in offspring at 8‐year follow‐up.

**Trial Registration:**

ClinicalTrials.gov identifiers: NCT03259919, NCT00159536 and NCT01587378

## 1 Introduction

Polycystic ovary syndrome (PCOS) is an endocrinopathy affecting 10%–13% of women and involves oligo‐ or anovulation, insulin resistance, hyperandrogenism, polycystic ovaries and increased risk of pregnancy complications [1]. Women with PCOS have an altered immune response in the non‐pregnant state, characterised by increased inflammatory markers [2, 3]. The clinical effects of these immune alterations are not fully understood, but it has been reported that women with PCOS experience more autoimmune diseases [4], more asthma [5], and may have higher susceptibility to infections such as COVID‐19 [6, 7]. We have previously shown that the inflammatory state seen in PCOS persists in pregnancy and further involves an increase in serum cytokines, reflecting a more activated immune system [8].

Maternal PCOS status may affect the short‐ and long‐term health of the offspring. Infants of mothers with PCOS have more congenital anomalies, higher perinatal mortality, lower Apgar scores and more hospitalisations [9]. Long‐term, the children have poorer metabolic health and increased risk of hospitalisation due to upper and lower respiratory tract infections and asthma [10, 11].

Metformin is a glucose‐lowering drug commonly used for PCOS, as it improves clinical pregnancy rates and lowers androgen levels and insulin resistance [1]. We have previously demonstrated that metformin treatment throughout pregnancy in women with PCOS induces a broad immune response by increasing serum levels of multifunctional cytokines [12]. Studies in non‐pregnant individuals indicate that metformin treatment may lower the incidence of infections [13, 14], but it has not been determined whether this extends to pregnancy. However, metformin treatment in pregnancy has been shown to reduce the occurrence of preterm birth [15], and infections are known mediators for preterm birth [16].

Metformin crosses the placenta and might therefore directly affect the fetus [17]. Teratogenicity has not been reported [18], but metformin‐exposed offspring seem to have lower birth weight [19], larger head circumference at birth [20], and increased body mass index (BMI) and weight at long‐term follow‐up compared to offspring exposed to placebo or insulin in utero [21, 22, 23]. The need for more data on metformin's long‐term effects on offspring has been highlighted [24, 25].

This study aimed to evaluate the immunological effects of metformin by comparing the incidence of infections in pregnant women with PCOS randomised to metformin or placebo, as well as the long‐term risk of asthma, allergies and eczema in their offspring.

## 2 Methods

### 2.1 Study Population

This study includes two study populations: (1) pregnant women with PCOS from the PregMet [26] and PregMet2 [15] studies (Figure S1) and (2) offspring of women with PCOS (recruited from the Pilot study [27] and the PregMet study [26]) who participated in a follow‐up study termed the PedMet study [21] (Figure S2).

#### 2.1.1 Pregnant Women With PCOS

PregMet and PregMet2 are prospective, randomised, double‐blinded, multicenter trials that aimed to determine whether metformin reduced pregnancy complications in women with PCOS. The PregMet study (2005–2009) included 257 pregnant women with PCOS, with a total of 274 pregnancies (17 women participated twice). The PregMet2 study (2012–2017) included 487 pregnant women with PCOS. In both studies, women were randomised to daily doses of 2000 mg metformin or placebo from the first trimester to delivery. Primary and secondary outcomes, as well as inclusion and exclusion criteria in the original studies, are described in Table S1 and in the original publications [15, 26]. Women with misdiagnosed PCOS, early miscarriage or termination of pregnancy were excluded from the present study.

Clinical information was collected at inclusion (gestational weeks 5 to 12) and at study visits in gestational weeks 19 ± 1, 24 ± 1 (PregMet), 28 ± 1 (PregMet2), 32 ± 1 and 36 ± 1. Incidence of self‐reported maternal infections and use of antibiotics between the study visits, as well as infections at delivery and postpartum, were recorded as adverse events and registered in a structured manner at each study visit in the original studies. For the present study, maternal infections during pregnancy were categorised as viral, bacterial or fungal based on the most likely infectious agent. Binary summary variables on infections were constructed: women with one or more viral/bacterial/fungal/total infections during pregnancy and women with no viral/bacterial/fungal/total infections during pregnancy. All infections recorded at delivery and postpartum (fever, sepsis, endometritis, chorioamnionitis, wound infections and mastitis) were bacterial and required antibiotics. Women with baseline BMI < 25 kg/m^2^ were defined as normal weight, BMI ≥ 25 kg/m^2^ and < 30 kg/m^2^ as overweight, and BMI ≥ 30 kg/m^2^ as obese in accordance with WHO criteria [28].

#### 2.1.2 Offspring of Women With PCOS

The PedMet study (2014–2016) [21] is a follow‐up of offspring from the Pilot study [27] and the PregMet study that aimed to assess potential effects of in utero metformin exposure on offspring growth and anthropometric measures. The Pilot study (2000–2003) included 40 pregnant women with PCOS and had a similar design as the PregMet study, but the women received a lower dose of metformin—1700 mg daily, from inclusion until delivery [27]. Primary and secondary outcomes, as well as inclusion and exclusion criteria, can be found in Table S1 and in the original publication [27]. Some women participated in the original studies with two pregnancies, resulting in pairs of siblings in the follow‐up studies. Primary and secondary outcomes, inclusion and exclusion criteria for the PedMet study are described in Table S1, and parts of the data from this cohort have been published previously [21]. Those who did not attend the scheduled clinical examination were excluded from the present study.

Patient history of the offspring was collected during the clinical examination at around 8 years of age. This included information from the mothers on whether the offspring had been diagnosed with asthma or eczema. Although allergies were not systematically surveyed, offspring treated with antihistamines were classified as allergic based on reported medical treatments and indications. Asthma, allergies and eczema will be referred to as ‘allergic diseases’ for simplicity, unless otherwise specified.

No patients participated in the development of the studies, and no core outcome set was used as an outcome measure. Written informed consent was obtained from all participating women and from each child's parent or guardian before inclusion.

### 2.2 Data Processing and Statistical Analyses

All analyses were performed in RStudio (R 4.4.0). Missing data for infections at each study visit during pregnancy, at delivery and postpartum were imputed using the *k*‐nearest neighbour algorithm from the R‐package VIM [29]. Baseline variables on maternal age, blood pressure, BMI and parity were used for imputations; numeric variables were mean scaled before imputation, and the number of *k* that gave the best precision was chosen. Sensitivity analyses were performed without imputed data. There were no missing data on allergic diseases for the offspring. Maternal and offspring data were analysed both according to the intention‐to‐treat (ITT) and per‐protocol (PP) principle. ITT analyses included all participants, whereas PP analyses excluded dropouts and participants with poor adherence to study medication, defined as < 70% intake of study medication. PP analyses are presented as main results as they more accurately reflect the biological effects of metformin. ITT analyses are presented as Supporting Information.

Clinical outcomes of the pregnant women randomised to metformin or placebo and their offspring were compared using the Mann–Whitney *U* test for continuous variables, and the chi square or Fisher's exact test for categorical data. For the offspring, weight, height and BMI were converted to age‐ and gender‐adjusted *z*‐scores according to a Norwegian reference population [30].

Absolute risk differences with corresponding confidence intervals were calculated to provide an estimate of the total number of maternal infections during pregnancy, at delivery and postpartum in the metformin‐ and placebo‐treated groups, respectively. Logistic regression was used to compare the effect of metformin with that of placebo. We conducted two analyses: one unadjusted analysis and another with maternal BMI at inclusion as a covariate. Adjustment for maternal BMI was decided a priori as it is considered an important risk factor for infections in pregnancy [31]. Additionally, we investigated BMI as an effect modifier by including maternal BMI and the interaction between BMI and metformin treatment as covariates. Adjustments for interdependency between women participating with two pregnancies were added to the model as a random intercept; however, this resulted in poor fit (standard deviations close to 0 for the random intercept), possibly due to the small number of such cases. Consequently, adjustments for interdependency between these women were not included in the final model.

Absolute risk differences with corresponding confidence intervals were calculated to estimate the incidence of allergic disease in offspring exposed to metformin or placebo in utero. Logistic regression was used to compare the effect of metformin on allergic diseases in the offspring with that of placebo. Adjustments for interdependency between siblings were not included in the final model due to poor fit. Mediator analyses were performed to evaluate whether offspring BMI *z*‐score at follow‐up and maternal infection status in pregnancy influenced the incidence of allergic diseases in the offspring. For this, we evaluated whether metformin exposure was associated with the potential mediator variable, and if so, whether adding the mediator to the regression analysis between metformin exposure and allergic disease changed the results.

### 2.3 Use of Artificial Intelligence

In the preparation of this manuscript, we used the artificial intelligence language model Microsoft Copilot to enhance the quality of the text. For each paragraph, we applied the following prompt: ‘We are submitting a manuscript to a scientific article. Please provide specific feedback on spelling, grammar, structure and clarity. Please come up with questions and suggestions that helps improve the quality of the manuscript.’ We critically reviewed and edited the output before incorporating it into the manuscript. We take full responsibility for the content of the manuscript.

## 3 Results

### 3.1 Study Population

#### 3.1.1 Pregnant Women With PCOS (PregMet and PregMet2 Studies)

The PregMet and PregMet2 studies included 744 pregnant women with PCOS with a total of 761 pregnancies (Figure S1). Six women were excluded, leaving 738 pregnant women with 755 pregnancies for the ITT analyses. Of these, 377 were randomised to metformin, and 378 were randomised to placebo. After excluding dropouts (*N* = 57) and women with poor adherence to the study medication (*N* = 50), 634 women with 648 pregnancies remained for the PP analyses. Among these, 316 were randomised to metformin and 332 to placebo. Women randomised to metformin had fewer preterm births, as reported previously [15] (Table 1; and Table S2). Otherwise, they were comparable to the women randomised to placebo.

**TABLE 1:** Maternal baseline characteristics and pregnancy outcomes of women with PCOS by metformin or placebo randomization (per‐protocol analysis, PregMet and PregMet2 studies).

	Metformin (*N* = 316)	Placebo (*N* = 332)
Maternal characteristics		
Age (years)	30 (27–33)	30 (27–33)
BMI (kg/m^2^)	28.3 (23.8–33.3)	26.7 (23.3–31.2)
Nulliparous	184 (58)	190 (57)
SBP (mmHg)	117 (110–124)	115 (107–122)
DBP (mmHg)	73 (67–79)	71 (65–78)
Smoking	16 (5.1)	16 (4.8)^1^
Metformin use at conception	94 (30)	83 (25)
Asthma	23 (7.3)	36 (11)
Allergy	6 (1.9)	8 (2.4)
Eczema	2 (0.6)	2 (0.6)
PCOS phenotype		
Hyperandrogenic	242 (76)^1^	252 (76)^3^
Normoandrogenic	73 (23)^1^	77 (23)^3^
Pregnancy outcomes^a^		
Late miscarriage	2 (0.6)	7 (2.1)
Preterm birth	12 (3.8)	28 (8.4)
Preeclampsia	17 (5.4)	21 (6.3)
Gestational diabetes mellitus	120 (41)^26^	129 (40)^13^
Mode of delivery^a^		
Vaginal delivery	217 (69)	231 (70)
Vacuum extraction	31 (9.8)	33 (9.9)
Forceps	3 (0.9)	3 (0.9)
Caesarean section	65 (21)	65 (20)
Characteristics at delivery^a^		
Gestational age (days)	279 (272–285)	280 (271–286)
Birth weight (g)	3560 (3250–3960)^1^	3580 (3215–3870)^4^
Birth length (cm)	50 (49–52)^6^	50 (49–52)^11^
Placental weight (g)	650 (580–760)^33^	650 (570–740)^45^
Fetal sex, female	155 (49)^1^	160 (49)^3^

*Note:* Continuous variables are reported as median (25th–75th percentile)^*m*^, and categorical variables as *N* (%)^*m*^, where *m* is the number of missing data points.

Abbreviations: BMI, body mass index; DBP, diastolic blood pressure; PCOS, polycystic ovary syndrome; SBP, systolic blood pressure.

^a^ Comparisons were made by Mann–Whitney *U* test for continuous variables, and the chi squared or Fisher's exact test for categorical data. There were no significant between‐group differences except for preterm birth (*p* = 0.014).

#### 3.1.2 Offspring of Women With PCOS (PedMet Study)

Of the 293 offspring of women with PCOS who had participated in the Pilot study or the PregMet study and were invited to the follow‐up, 159 (54%) agreed to take part in the PedMet study (Figure S2). One child did not attend the clinical examination and was therefore excluded, leaving 158 offspring for the ITT analyses, with 80 exposed to metformin and 78 exposed to placebo in utero. Among these, there were 11 pairs of siblings. After excluding 13 offspring whose mothers had poor compliance to study medication, 145 remained for PP analyses, with 74 exposed to metformin and 71 to placebo in utero. Among these, there were 10 pairs of siblings. At the time of follow‐up in the PedMet study, the offspring were approximately 8 years old (Table 2; and Table S3). Offspring exposed to metformin in utero had a larger head circumference at birth and a higher weight *z*‐score and BMI *z*‐score at follow‐up compared to those exposed to placebo, as previously reported [20, 21]. Maternal baseline characteristics and pregnancy outcomes for offspring participating in the PedMet study were comparable between the metformin and placebo groups (Tables S4 and S5). Moreover, maternal baseline characteristics, pregnancy outcomes and offspring birth characteristics were similar between participants and non‐participants in the PedMet study (Table S6).

**TABLE 2:** Characteristics at birth and at 8‐year follow‐up for offspring exposed to metformin or placebo in utero (per‐protocol analysis, PedMet study).

	Metformin (*N* = 74)	Placebo (*N* = 71)	*p*
Characteristics at birth			
Gestational age (days)	281 (272–286)	279 (268–285)	0.4
Birth weight (g)	3550 (3320–3930)	3500 (3090–3940)	0.5
Birth weight (*z*‐score)	−0.15 (−0.85–0.60)	−0.08 (−0.62–0.68)	0.4
Birth length (cm)	50 (49–51)	50 (48–52)^1^	0.8
Birth length (*z*‐score)	−0.57 (−1.39–0.29)	−0.28 (−1.19–0.51)^1^	0.3
Head circumference (cm)	36 (35–37)	35 (34–36)	**0.01**
Head circumference (*z*‐score)	0.33 (−0.30–0.93)	0.15 (−0.71–0.88)	0.2
Sex female	36 (49)	39 (55)	0.4
Characteristics at follow‐up			
Age (years)	7.9 (6.6–8.8)	7.8 (6.9–9.1)	0.8
Weight (kg)	30 (24–38)^2^	28 (24–33)	0.3
Weight (*z*‐score)	0.48 (−0.28–1.65)^2^	0.19 (−0.62–0.89)^1^	**0.03**
Height (cm)	129 (122–139)^2^	129 (122–137)	0.7
Height (*z*‐score)	0.22 (−0.58–0.91)^2^	0.01 (−0.64–0.50)^1^	0.2
BMI (kg/m^2^)	17.3 (15.6, 20.3)^2^	16.3 (15.1–19.1)	0.09
BMI (*z*‐score)	0.50 (−0.31–1.66)^2^	0.00 (−0.60–0.97)^1^	**0.05**

*Note:* Continuous variables are reported as median (25th–75th percentile)^*m*^, and categorical variables as *N* (%)^*m*^, where *m* is the number of missing data points. Comparisons were made by Mann–Whitney *U* test for continuous data, and the chi square or Fisher's exact test for categorical data. Significant *p*‐values are shown in bold.

Abbreviation: BMI, body mass index.

### 3.2 Incidence of Infections During Pregnancy, at Delivery and Postpartum in Women With PCOS Treated With Metformin or Placebo

Metformin treatment led to a significant reduction in maternal infections during pregnancy in women with PCOS (Table 3). Of the women treated with metformin (*N* = 316), 42% experienced one or more infections during pregnancy, compared to 52% of those treated with placebo (*N* = 332) (OR = 0.68, 95% CI: 0.50–0.93). There were fewer viral infections throughout pregnancy in women treated with metformin (OR = 0.71, 95% CI: 0.51–0.99). The most common viral infection was respiratory tract infections, and women treated with metformin appeared to have fewer respiratory tract infections compared to women treated with placebo (26% vs. 33%, respectively). Bacterial infections during pregnancy, at delivery and postpartum, as well as fungal infections and use of antibiotics during pregnancy, did not differ between women treated with metformin and placebo. Adjustment for baseline BMI did not influence the results, and absolute risk differences were consistent with the main findings (Table 3).

**TABLE 3:** Incidence of infections during pregnancy, delivery and postpartum in women with PCOS randomised to metformin or placebo (per‐protocol analysis, PregMet and PregMet 2 studies).

	Metformin (*N* = 316)	Placebo (*N* = 332)	ARD (95% CI)	Crude analysis		Adjusted analysis*	
				Odds ratio (95% CI)	*p*	Odds ratio (95% CI)	*p*
**During pregnancy**							
Viral infections	94 (30)	124 (37)	−0.08 (−0.15 to −0.004)	0.71 (0.51–0.99)	**0.041**	0.71 (0.51–0.99)	**0.042**
Respiratory tract infections	81 (26)^2^	111 (33)					
Gastroenteritis	19 (6.1)^2^	25 (7.5)					
Other viral infections^a^	5 (1.6)^2^	3 (0.9)					
Bacterial infections	52 (16)	66 (20)	−0.03 (−0.09 to 0.03)	0.79 (0.53–1.18)	0.3	0.79 (0.52–1.18)	0.2
Respiratory tract infections	14 (4.5)^2^	19 (5.7)					
Urinary tract infections	30 (9.6)^2^	43 (13)					
Other bacterial infections^b^	10 (3.2)^2^	8 (2.4)					
Use of antibiotics	51 (16)^2^	64 (19)					
Fungal infections	12 (3.8)	13 (3.9)	−0.001 (−0.03 to 0.03)	0.97 (0.43–2.17)	> 0.9	0.97 (0.43–2.18)	> 0.9
Vaginal fungal infections	10 (3.2)^2^	11 (3.3)					
Other fungal infections^c^	2 (0.6)^2^	2 (0.6)					
Viral, bacterial and fungal infections	133 (42)	171 (52)	−0.09 (−0.17 to −0.02)	0.68 (0.50–0.93)	**0.017**	0.69 (0.50–0.94)	**0.019**
**At delivery or postpartum**							
Total infections	25 (7.9)	22 (6.6)	0.01 (−0.03 to 0.05)	1.21 (0.67–2.21)	0.5	1.18 (0.65–2.16)	0.6
Fever	11 (3.5)	10 (3)					
Other infections^d^	16 (5.1)	12 (3.6)					

*Note:* Categorical variables are reported as *N* (%)^*m*^, where *m* is the number of missing data points. Comparisons were made by logistic regression. Significant *p*‐values are shown in bold. All *p*‐values are nominal without adjustment for multiple testing.

Abbreviations: ARD, absolute risk differences; CI, confidence interval; PCOS, polycystic ovary syndrome.

^a^ Chickenpox (*N* = 1) and herpes simplex (*N* = 4) in the metformin group. Herpes simplex (*N* = 1), herpes zoster (*N* = 1) and viral meningitis (*N* = 1) in the placebo group.

^b^ Bacterial vaginosis (*N* = 1), otitis (*N* = 1), conjunctivitis (*N* = 1), skin infection (*N* = 2), dental abscess (*N* = 4) and unspecified infection (*N* = 1) in the metformin group. Bacterial breast infection (*N* = 1), chlamydia (*N* = 1), otitis (*N* = 2), conjunctivitis (*N* = 1), pyelonephritis (*N* = 1), tick‐borne infection (*N* = 1) and unspecified infection (*N* = 1) in the placebo group.

^c^ Oral candidiasis (*N* = 1) and breast candidiasis (*N* = 1) in the metformin group. Oral candidiasis (*N* = 2) in the placebo group.

^d^ Chorioamnionitis (*N* = 5), sepsis (*N* = 1), wound infection after caesarean section (*N* = 4), vulvar wound infection (*N* = 1) and mastitis (*N* = 5) in the metformin group. Chorioamnionitis (*N* = 5), wound infection after caesarean section (*N* = 5) and mastitis (*N* = 2) in the placebo group.

* Adjusted for maternal baseline body mass index.

While women treated with metformin had fewer viral infections during pregnancy compared to the placebo group in all BMI categories, the difference was only statistically significant for obese women in subgroup analysis (OR = 0.52, 95% CI: 0.30–0.89) (Table S7). Obese women also tended to have fewer overall infections in pregnancy following metformin treatment (OR = 0.62, 95% CI: 0.37–1.04). However, the interaction between metformin treatment and baseline BMI did not reach significance (Table S7).

Results in the ITT and PP analyses were comparable (Tables S8 and S9; and Table 3 and Table S7, respectively). Similar results were achieved in sensitivity analyses excluding women with missing data on infection status (Tables S10 and S11).

### 3.3 Incidence of Allergic Diseases in Offspring Exposed to Metformin or Placebo In Utero

Offspring exposed to metformin in utero (*N* = 74) showed a significantly higher incidence of allergies and eczema at around 8 years of age compared to those exposed to placebo in utero (*N* = 71) (Table 4). Between birth and follow‐up, 18% of the metformin‐exposed offspring developed allergies, compared to 4.2% of the placebo‐exposed offspring (OR = 4.83, 95% CI: 1.47–21.8). Similarly, 35% of the metformin‐exposed offspring experienced eczema between birth and follow‐up, compared to 18% of the placebo‐exposed offspring (OR = 2.42, 95% CI: 1.14–5.33). The incidence of asthma did not differ (OR = 1.52, 95% CI: 0.59–4.13) (Table 4). Absolute risk differences were consistent with these findings (Table 4).

**TABLE 4:** Incidence of allergic diseases at 8‐year follow‐up in offspring exposed to metformin or placebo in utero (per‐protocol analysis, PedMet study).

	Metformin (*N* = 74)	Placebo (*N* = 71)	ARD (95% CI)	Crude analysis		Adjusted analysis*	
				Odds ratio (95% CI)	*p*	Odds ratio (95% CI)	*p*
Asthma	12 (16)	8 (11)	0.05 (−0.06 to 0.16)	1.52 (0.59–4.13)	0.4	—	—
Allergy	13 (18)	3 (4.2)	0.13 (0.04 to 0.23)	4.83 (1.47–21.8)	**0.018**	4.85 (1.46–22.1)	**0.019**
Eczema	26 (35)	13 (18)	0.17 (0.03 to 0.31)	2.42 (1.14–5.33)	**0.024**	2.54 (1.17–5.76)	**0.021**

*Note:* Categorical variables are reported as *N* (%). Comparisons were made by logistic regression. Significant *p*‐values are shown in bold. All *p*‐values are nominal without adjustment for multiple testing. —: Analysis not performed due to no association in crude analysis.

Abbreviations: ARD, absolute risk differences; CI, confidence interval.

* Body mass index *z*‐score was included as a covariate to examine whether it mediated the relationship observed in crude analysis.

Neither offspring BMI *z*‐score nor maternal infections during pregnancy affected the association between in utero metformin exposure and the risk of allergies and eczema in offspring, indicating that they were not mediators of the metformin effect (Table 4 and Figure S3).

Results in the ITT and PP analyses were comparable (Table S12 and Table 4, respectively).

## 4 Discussion

### 4.1 Main Findings

By analysing data from two randomised controlled trials (RCTs) and one related follow‐up study, we provide new insights into the effects of metformin on immunological outcomes in pregnant women with PCOS and their offspring. Our findings demonstrate that metformin treatment throughout pregnancy resulted in fewer maternal infections, particularly viral infections, with the strongest effect observed in women with obesity. We further show that offspring exposed to metformin in utero had more allergies and eczema at around 8 years of age. No relationship was found between the decreased incidence of maternal infections, offspring BMI *z*‐score, and the development of allergies and eczema.

### 4.2 Interpretation

This is the first study to show that metformin reduces infections during pregnancy in women with PCOS, a finding supported by similar effects in other populations [13, 14, 32]. An RCT in non‐pregnant women with PCOS reported that those treated with metformin had significantly fewer urinary tract infections [14]. A placebo‐controlled trial in patients with inflammatory diseases found that metformin significantly reduced pneumonia and the overall rate of moderate‐to‐severe infections [13]. Three previous RCTs on pregnant women with gestational diabetes or type 2 diabetes found no difference in the incidence of maternal infections (reported as adverse events) [33, 34, 35]. However, these trials primarily focused on severe infections or compared metformin to other medications rather than placebo, reducing the comparability to the present study. We found no association between metformin treatment and bacterial infections either during pregnancy, at delivery or postpartum. This is supported by an RCT that found no difference in bacterial infections at delivery when comparing metformin treatment to placebo in overweight and obese pregnant women [36].

To our knowledge, no other studies have investigated the long‐term effects of in utero metformin exposure on allergic diseases in offspring. Consequently, we are the first to report an association between metformin exposure in utero and an increased incidence of allergies and eczema in childhood. Increased birthweight and BMI in childhood have been associated with the development of allergic diseases [37, 38]. We did not observe any difference in birthweight between metformin‐ and placebo‐exposed offspring, making it less likely that birthweight explains our findings. Moreover, the increased BMI *z*‐score in metformin‐exposed offspring at follow‐up was not a mediator of allergic diseases in the present study, indicating that the effect of metformin on allergies and eczema was independent of BMI. Metformin did not affect the incidence of asthma at follow‐up, which is in accordance with an 8‐year follow‐up study of the mothers from the Pilot study and the PregMet study showing no effect of metformin on respiratory health [5]. Nevertheless, the negative health effects on offspring demonstrated in the present study raise concerns about the use of metformin during pregnancy.

Metformin may impact maternal susceptibility to infections through metabolic and immunological pathways. The reduction of maternal infections observed in the present study was most pronounced in obese women, suggesting significant metabolic effects of metformin. A recent review proposes that metformin might reduce or ameliorate infections through its anti‐hyperglycemic properties [32]. As insulin resistance and hyperglycaemia are cornerstones in PCOS [1], it is likely that the glucose‐lowering effects of metformin might help prevent infections. However, previous analyses from our cohort did not reveal any effect of metformin on glucose homeostasis in pregnancy [39], indicating that additional mechanisms are involved. Jiménez‐Escutia et al. found reduced production of cytokines in placentas exposed to both hyperglycaemia and a pathogen, suggesting a weakened placental response to pathogens following hyperglycaemia [40]. We have previously demonstrated that metformin induces a broad maternal immune mobilisation by increasing multifunctional serum cytokines [12]. Although the design of the present study does not allow us to directly answer this question, it is tempting to speculate whether this immune mobilisation might be protective against infections during pregnancy.

Maternal health during pregnancy, including exposure to microbes, impacts the offspring and primes the development of the fetal immune system [41, 42]. Therefore, it is likely that metformin‐induced changes in the mother can affect the child and contribute to an increased risk of allergic diseases. Maternal infections during pregnancy have been proposed as both a risk factor [43] and a protective factor [44, 45] for the development of allergic diseases in the offspring. In the present study, metformin reduced the incidence of maternal infections during pregnancy while it increased the incidence of eczema and allergies in the offspring, suggesting that maternal infections might act as a protective factor. However, the lack of association between maternal infections and offspring outcomes in our mediator analysis prevents us from drawing a definitive conclusion.

Allergic diseases are mainly characterised by a T‐helper cell 2 response [46], and therefore it is interesting that the T‐helper 2 cytokines eotaxin and IL‐4 were increased in maternal serum following metformin treatment [12]. A correlation between maternal eotaxin levels and cord blood eotaxin levels has been observed, suggesting that eotaxin might cross the placenta and affect the fetus [47]. However, we do not know how metformin‐induced changes in maternal serum cytokines translate to the offspring, as studies on immune markers in offspring following metformin exposure in utero are lacking. This is a highly interesting field for future research.

### 4.3 Strengths and Limitations

A main strength of the present study is that the study cohort originated from randomised controlled trials, minimising the influence of confounders. The RCTs were conducted according to good clinical practice with strict randomisation and masking procedures [15, 26, 27], a large sample size, and a longitudinal design. We conducted both ITT and PP analyses and were therefore able to analyse the effects in women who had taken metformin as prescribed. A limitation is that the included studies were not originally designed to investigate the relationship between metformin and infection or allergic diseases. Infections during pregnancy were self‐reported and classified into categories based on the most likely pathogen determined by clinical signs, symptoms and treatment, but we were unable to determine the exact pathogen. Information on maternal infections was not collected in the Pilot study; therefore, these women could not be included in the analyses of maternal infections during pregnancy, delivery or postpartum. Asthma, eczema and allergies were doctor‐diagnosed, but reported by the mother, and not verified with adequate medical tests. Moreover, asthma, eczema and allergies are heterogeneous diseases, and we lacked information about specific subtypes. Offspring from the Pilot study and the PregMet study, but not the PregMet2 study, have been followed up, and the limited proportion of offspring participating in the follow‐up study may have introduced selection bias. However, comparison of participants vs. non‐participants revealed no significant differences, suggesting that the risk of selection bias is minimal. Furthermore, the randomised design ensures that inaccuracies are equally distributed in both groups.

## 5 Conclusion

In conclusion, metformin treatment during pregnancy in women with PCOS reduced maternal infections but increased the long‐term risk of allergies and eczema in their offspring. This suggests that metformin induces immunological changes both in the mother during pregnancy and in the offspring over the long term. More research is needed to understand the maternal immunological changes during pregnancy and how they affect the offspring. The adverse effects observed in the offspring highlight the need for caution when considering metformin treatment during pregnancy.

## Author Contributions

M.R., J.E.S., G.F.G., A.‐C.I. and E.V. designed the present study. E.V., T.S.L. and L.G.E.H. were principal investigators and collected the data for the Pilot study, the PregMet study, the PregMet2 study and the PedMet study, respectively. M.R., J.E.S. and A.H.J. prepared, analysed, and interpreted the data for the present study with supervision from G.F.G., A.‐C.I. and E.V. M.R. wrote the manuscript. All authors were involved in revising the manuscript and approved the final version for publication.

## Ethics Statement

All studies were approved by the Regional Committee for Medical and Health Research Ethics (REC) (Pilot: No. 86‐99, 28 March 2000; PregMet: No. 145.04, 23 September 2004; PregMet2: No. 2011/1434, 9 January 2014; PedMet: No. 2014/96, 30 May 2014). The declaration of Helsinki was followed throughout the studies. The studies were conducted according to principles of ‘Good Clinical Practice’, and the trials are registered at ClinicalTrials.gov (Pilot and follow‐up offspring: NCT03259919, PregMet and PedMet studies: NCT00159536, PregMet2: NCT01587378).

## Conflicts of Interest

The authors declare no conflicts of interest.

## Supporting information


**Figure S1:** Flowchart of inclusion, randomization and exclusions of pregnant women with PCOS randomised to metformin or placebo throughout pregnancy. PCOS, polycystic ovary syndrome.

**Figure S2:** Flowchart of inclusion, randomization and exclusions of offspring exposed to metformin or placebo in utero.

**Figure S3:** Conceptual diagram of mediation analysis between metformin exposure and offspring allergy (A) or eczema (B), with BMI *z*‐score or maternal infections as possible mediators (per‐protocol analysis). Solid lines indicate significant relationships between predictor and outcome variables. Odds ratios or coefficients from logistic regression analyses are provided for the various relationships. If there was no significant relationship between the predictor (metformin exposure) and the mediator (BMI *z*‐score or maternal infections), a full mediation analysis was not conducted. If a significant association was found between the predictor and mediator, the mediator was included as a covariate to determine if it altered the relationship between the predictor and outcome. BMI, body mass index; CI, confidence interval; OR, odds ratio; *p*, *p*‐value.

**Table S1:** General information from the original studies used for selection of pregnant women with PCOS and offspring to the present study.

**Table S2:** Maternal baseline characteristics and pregnancy outcomes of women with PCOS by metformin or placebo randomization (intention‐to‐treat analysis, PregMet and PregMet2 studies).

**Table S3:** Characteristics at birth and at 8‐year follow‐up for offspring exposed to metformin or placebo in utero (intention‐to‐treat analysis, PedMet study).

**Table S4:** Baseline characteristics and pregnancy outcomes of mothers whose offspring participated in the PedMet study by metformin or placebo randomization (per‐protocol analysis).

**Table S5:** Baseline characteristics and pregnancy outcomes of mothers whose offspring participated in the PedMet study by metformin or placebo randomization (intention‐to‐treat analysis).

**Table S6:** Maternal and offspring characteristics of participants and non‐participants in the PedMet study.

**Table S7:** Incidence of infections during pregnancy, delivery and postpartum in women with PCOS randomised to metformin or placebo, stratified by maternal baseline BMI (per‐protocol analysis, PregMet and PregMet2 studies).

**Table S8:** Incidence of infections during pregnancy, delivery and postpartum in women with PCOS randomised to metformin or placebo (intention‐to‐treat analysis, PregMet and PregMet 2 studies).

**Table S9:** Incidence of infections during pregnancy, delivery and postpartum in women with PCOS randomised to metformin or placebo, stratified by maternal baseline BMI (intention‐to‐treat analysis, PregMet and PregMet2 studies).

**Table S10:** Incidence of infections during pregnancy, delivery and postpartum in women with PCOS randomised to metformin or placebo without imputed data (per‐protocol analysis, PregMet and PregMet2 studies).

**Table S11:** Incidence of infections during pregnancy, delivery and postpartum in women with PCOS randomised to metformin or placebo without imputed data (intention‐to‐treat analysis, PregMet and Pregmet2 studies).

**Table S12:** Incidence of allergic diseases at 8‐year follow‐up in offspring exposed to metformin or placebo in utero (intention‐to‐treat analysis, PedMet study).

## Data Availability

The data that support the findings of this study are available from the corresponding author upon reasonable request.
