# Supplementary material for: Metformin Treatment in PCOS Pregnancies Reduces Maternal Infections and Increases the Risk of Allergies and Eczema in the Offspring: Post Hoc Analyses of Two Randomised Controlled Trials and One Follow‐Up Study
Source: BJOG. 2025 Aug 11;132(12):1823–32. doi: 10.1111/1471-0528.18320 (PMC12501709; doi:10.1111/1471-0528.18320)
Supplement: Supplementary file 1 — Figure S1: Flowchart of inclusion, randomization and exclusions of pregnant women with PCOS randomised to metformin or placebo throughout pregnancy. PCOS, polycystic ovary syndrome. [file BJO-132-1823-s001.docx]

**
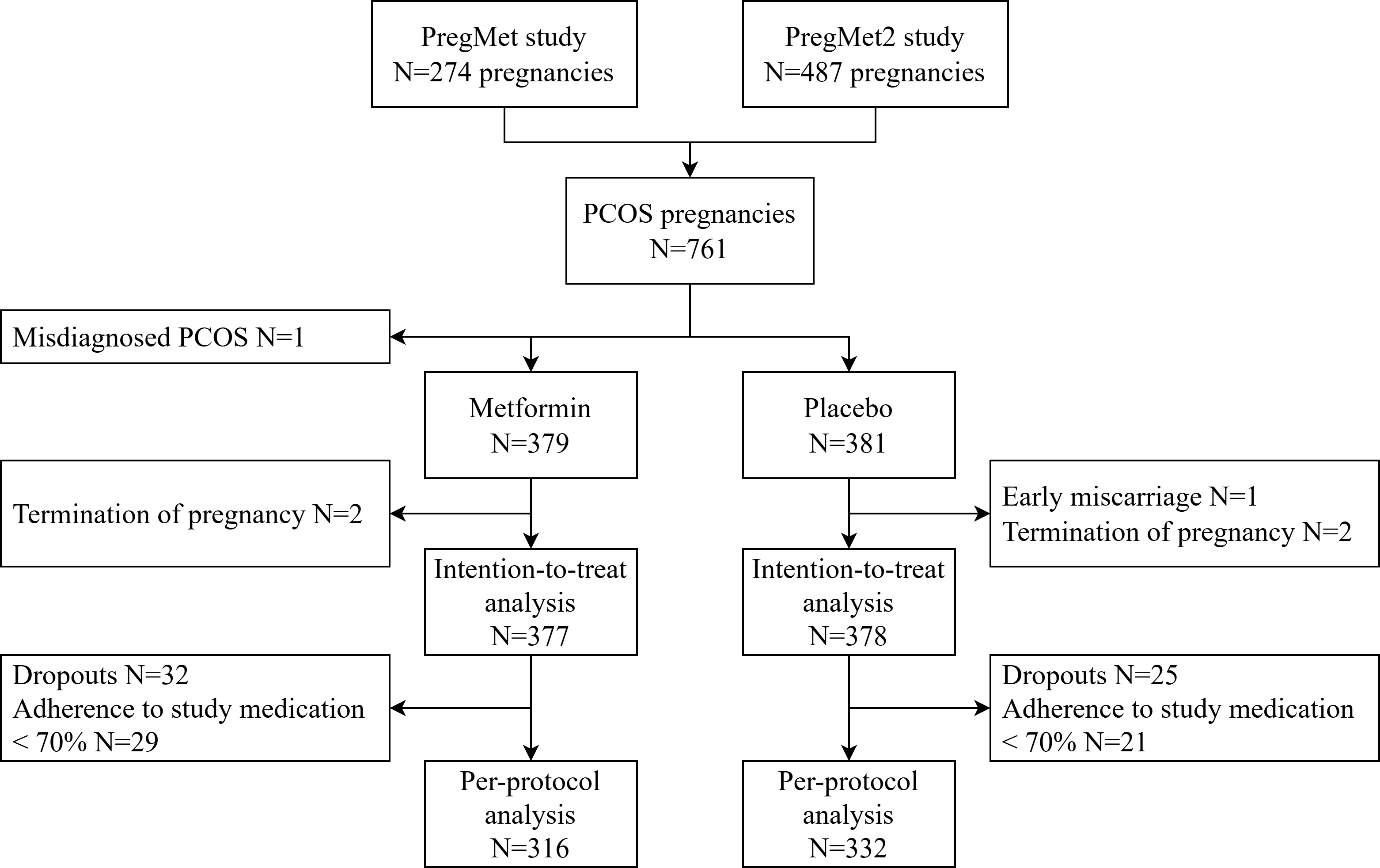
Figure S1:** Flowchart of inclusion, randomization, and exclusions of pregnant women with PCOS randomized to metformin or placebo throughout pregnancy.

Abbreviations: PCOS, polycystic ovary syndrome.
