# Supplementary material for: Metformin Treatment in PCOS Pregnancies Reduces Maternal Infections and Increases the Risk of Allergies and Eczema in the Offspring: Post Hoc Analyses of Two Randomised Controlled Trials and One Follow‐Up Study
Source: BJOG. 2025 Aug 11;132(12):1823–32. doi: 10.1111/1471-0528.18320 (PMC12501709; doi:10.1111/1471-0528.18320)
Supplement: Supplementary file 2 — Figure S2: Flowchart of inclusion, randomization and exclusions of offspring exposed to metformin or placebo in utero. [file BJO-132-1823-s008.docx]

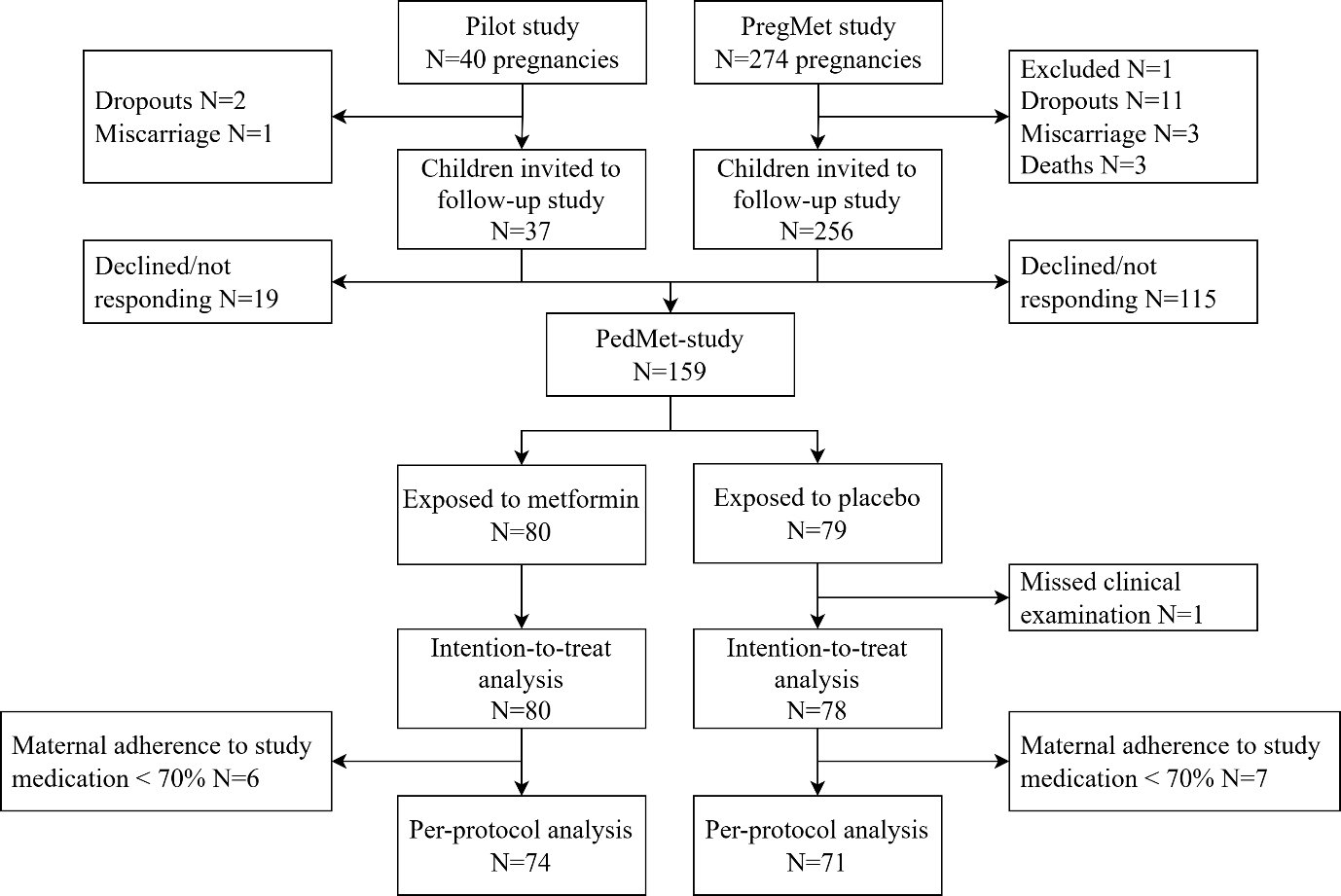


**Figure S2:** Flowchart of inclusion, randomization, and exclusions of offspring exposed to metformin or placebo in utero.
