# Supplementary material for: Metformin Treatment in PCOS Pregnancies Reduces Maternal Infections and Increases the Risk of Allergies and Eczema in the Offspring: Post Hoc Analyses of Two Randomised Controlled Trials and One Follow‐Up Study
Source: BJOG. 2025 Aug 11;132(12):1823–32. doi: 10.1111/1471-0528.18320 (PMC12501709; doi:10.1111/1471-0528.18320)
Supplement: Supplementary file 3 — Figure S3: Conceptual diagram of mediation analysis between metformin exposure and offspring allergy (A) or eczema (B), with BMI z‐score or maternal infections as possible mediators (per‐protocol analysis). Solid lines indicate significant relationships between predictor and outcome variables. Odds ratios or coefficients from logistic regression analyses are provided for the various relationships. If there was no significant relationship between the predictor (metformin exposure) and the mediator (BMI z‐score or maternal infections), a full mediation analysis was not conducted. If a significant association was found between the predictor and mediator, the mediator was included as a covariate to determine if it altered the relationship between the predictor and outcome. BMI, body mass index; CI, confidence interval; OR, odds ratio; p, p‐value. [file BJO-132-1823-s007.docx]

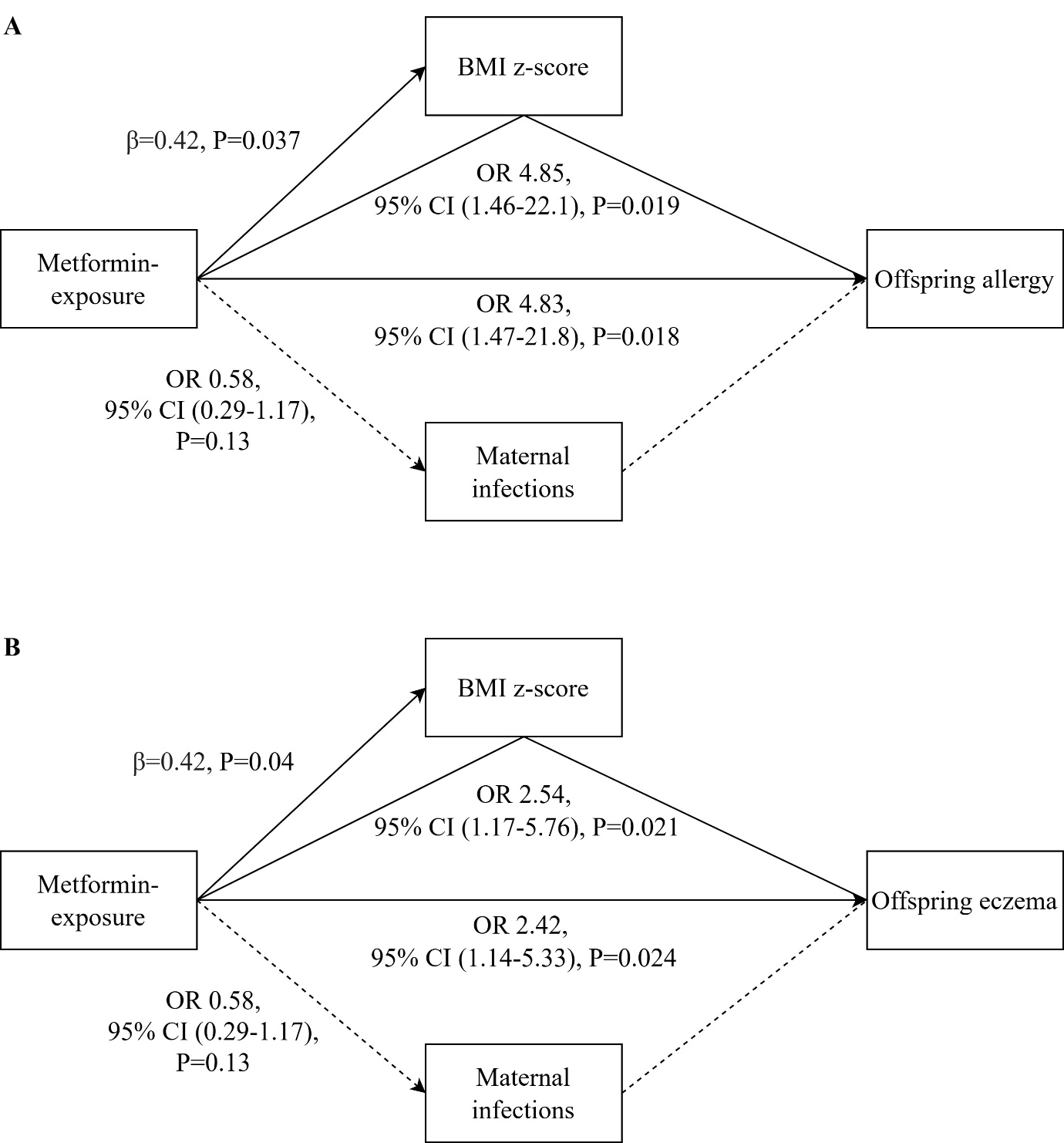


**Figure S3:** Conceptual diagram of mediation analysis between metformin exposure and offspring allergy (A) or eczema (B), with BMI z-score or maternal infections as possible mediators (per-protocol analysis). Solid lines indicate significant relationships between predictor and outcome variables. Odds ratios or coefficients from logistic regression analyses are provided for the various relationships. If there was no significant relationship between the predictor (metformin exposure) and the mediator (BMI z-score or maternal infections), a full mediation analysis was not conducted. If a significant association was found between the predictor and mediator, the mediator was included as a covariate to determine if it altered the relationship between the predictor and outcome.

Abbreviations: BMI, body mass index; CI, confidence interval; OR, odds ratio; P, P-value.
