# Supplementary material for: Metformin Treatment in PCOS Pregnancies Reduces Maternal Infections and Increases the Risk of Allergies and Eczema in the Offspring: Post Hoc Analyses of Two Randomised Controlled Trials and One Follow‐Up Study
Source: BJOG. 2025 Aug 11;132(12):1823–32. doi: 10.1111/1471-0528.18320 (PMC12501709; doi:10.1111/1471-0528.18320)
Supplement: Supplementary file 4 — Table S1: General information from the original studies used for selection of pregnant women with PCOS and offspring to the present study. [file BJO-132-1823-s013.docx]

**Table S1: General information from the original studies used for selection of pregnant women with PCOS and offspring to the present study**

|  | *Pregnant women with PCOS* | | | *Offspring* |
| --- | --- | --- | --- | --- |
|  | **Pilot** (1) | **PregMet** (2) | **PregMet2** (3) | **PedMet** (4) |
| **Participants** | N=40 | N=257 pregnant women  with 274 pregnancies | N=487 | N=18 (Offspring from the Pilot study, unpublished)  N=141 (Offspring from the PregMet study) |
| **Intervention** | Metformin (1700 mg) vs. placebo from first trimester to delivery | Metformin (2000 mg) vs. placebo from first trimester to delivery | Metformin (2000 mg) vs. placebo from first trimester to delivery | Exposure to metformin (1700 or 2000 mg) or placebo in utero |
| **Primary outcomes** | Serum levels of DHEAS, androstenedione, testosterone, SHBG, and FTI | PE, GDM, PD, and a composite of these three diagnoses | Composite incidence of LM and PD | BMI z-score |
| **Secondary outcomes** | Incidence of pregnancy complications and outcome | Weight, BP, HR, mode and length of delivery | GDM, PE, HT in pregnancy, Tx with vaginal progesterone to prevent imminent PD, and vaginal bleeding/admission hospital during pregnancy.  Neonatal secondary outcomes: Admission NICU and total number of days in NICU | Waist-to-height ratio/waist circumference/ weight/height/head circumference z-scores, muscle mass (kg), body fat (kg and %), adiponectin, ALAT, cholesterol (total, HDL, non-HDL), fasting glucose, HbA1c, insulin C-peptide, HOMA2-IR, HR, BP, IOTF BMI cutoffs, and cardiometabolic phenotypes |
| **Inclusion criteria** | (1) Diagnosis of PCOS before the actual pregnancy  (2) Age 18-40 years  (3) GW 5-12  (4) Singleton, viable fetus shown on ultrasonography | (1) PCOS diagnosed according to the Rotterdam criteria (5)  (2) Age 18–45 years  (3) GW 5-12  (4) Singleton, viable fetus shown on ultrasonography | (1) PCOS diagnosed according to the Rotterdam criteria (5)  (2) Age 18–45 years  (3) GW 6-12^+6^  (4) Pregnant by any mode of conception  (5) Singleton, viable fetus shown on ultrasonography  (4) Minimum 7 days washout of metformin (if used before inclusion)  (5) Ability to communicate in a Scandinavian language or English | (1) Mother included in the Pilot study or the PregMet study (see inclusion criteria for the respective studies)  (2) Written, informed consent from each child’s parent or guardian |
| **Exclusion criteria** | (1) Known liver failure  (2) Creatinine >130 mmol/l  (3) Known alcohol abuse  (4) Previously known DM  (5) Fasting plasma glucose >5.6 mmol/l  (6) Tx with oral glucocorticoids/drugs known to interfere with metformin | (1) ALAT > 90 IU/l  (2) Serum creatinine concentration >1.70 mg/dl  (3) Known alcohol abuse  (4) Previously known DM  (5) Fasting serum glucose >126 mg/dl at inclusion  (6) Tx with oral glucocorticoids/drugs known to interfere with metformin | (1) Known liver failure  (2) Known kidney failure  (3) Known alcohol/drug abuse  (4) Previously known DM  (5) Conditions that could induce tissue hypoxia  (6) Known hypersensitivity to metformin  (7) Tx with drugs known to interfere with metformin  (8) Breastfeeding  (9) Unsuitable for participation for other reasons | (1) Mother excluded in the Pilot study or the PregMet study (see exclusion criteria for the respective studies) |
| **Approvals** | REC No. 86-99 and ClinicalTrials.gov (NCT03259919) | REC No. 145.04 and ClinicalTrials.gov (NCT00159536) | REC No. 2011/1434 and ClinicalTrials.gov (NCT01587378) | REC No. 2014/96 and ClinicalTrials.gov  (Pilot offspring: NCT03259919,  PregMet offspring: NCT00159536) |
| Abbreviations: ALAT, alanine transaminase; BMI, body mass index; BP, blood pressure; DHEAS, dehydroepiandrosterone sulphate; DM, diabetes mellitus; FTI, free testosterone index; GDM, gestational diabetes mellitus; GW, gestational week; HbA1c, hemoglobin A1c; HDL, high-density lipoprotein; HOMA2-IR, homeostasis model assessment 2 for insulin resistance; HR, heart rate; HT, hypertension; IOTF, international obesity task force; LM, late miscarriage; NICU, neonatal intensive care unit; PCOS, polycystic ovary syndrome; PE, preeclampsia; PD, preterm delivery; REC, Regional Committee for Medical and Health Research Ethics; SHBG, sex hormone-binding globulin, Tx; treatment. | | | | |

**References**

1. Vanky E, Salvesen KA, Heimstad R, Fougner KJ, Romundstad P, Carlsen SM. Metformin reduces pregnancy complications without affecting androgen levels in pregnant polycystic ovary syndrome women: results of a randomized study. Hum Reprod. 2004 Aug;19(8):1734–40.

2. Vanky E, Stridsklev S, Heimstad R, Romundstad P, Skogøy K, Kleggetveit O, et al. Metformin versus placebo from first trimester to delivery in polycystic ovary syndrome: a randomized, controlled multicenter study. J Clin Endocrinol Metab. 2010 Dec;95(12):E448-455.

3. Løvvik TS, Carlsen SM, Salvesen Ø, Steffensen B, Bixo M, Gómez-Real F, et al. Use of metformin to treat pregnant women with polycystic ovary syndrome (PregMet2): a randomised, double-blind, placebo-controlled trial. The Lancet Diabetes & Endocrinology. 2019 Apr 1;7(4):256–66.

4. Hanem LGE, Salvesen Ø, Juliusson PB, Carlsen SM, Nossum MCF, Vaage MØ, et al. Intrauterine metformin exposure and offspring cardiometabolic risk factors (PedMet study): a 5-10 year follow-up of the PregMet randomised controlled trial. Lancet Child Adolesc Health. 2019 Mar;3(3):166–74.

5. Rotterdam ESHRE/ASRM-sponsored PCOS consensus workshop group. Revised 2003 consensus on diagnostic criteria and long-term health risks related to polycystic ovary syndrome. Fertil Steril. 2004 Jan;81(1):19–25.
