# Supplementary material for: Metformin Treatment in PCOS Pregnancies Reduces Maternal Infections and Increases the Risk of Allergies and Eczema in the Offspring: Post Hoc Analyses of Two Randomised Controlled Trials and One Follow‐Up Study
Source: BJOG. 2025 Aug 11;132(12):1823–32. doi: 10.1111/1471-0528.18320 (PMC12501709; doi:10.1111/1471-0528.18320)
Supplement: Supplementary file 5 — Table S2: Maternal baseline characteristics and pregnancy outcomes of women with PCOS by metformin or placebo randomization (intention‐to‐treat analysis, PregMet and PregMet2 studies). [file BJO-132-1823-s011.docx]

**Table S2: Maternal baseline characteristics and pregnancy outcomes of women with PCOS by metformin or placebo randomization (intention-to-treat analysis, PregMet and PregMet2 studies)**

|  | **Metformin (N=377)** | **Placebo (N=378)** |
| --- | --- | --- |
| **Maternal characteristics** | | |
| Age (years) | 30 (26-32) | 30 (27-33) |
| BMI (kg/m^2^) | 27.7 (23.6-32.9) | 26.8 (23.3-31.5) |
| Nulliparous | 220 (58) | 208 (55) |
| SBP (mmHg) | 115 (109-123) | 115 (107-122) |
| DBP (mmHg) | 73 (67-79) | 71 (65-79) |
| Smoking | 22 (5.8) | 22 (5.8)^1^ |
| Metformin use at conception | 107 (28) | 95 (25) |
| Asthma | 26 (6.9) | 41 (11) |
| Allergy | 7 (1.9) | 13 (3.4) |
| Eczema | 2 (0.5) | 2 (0.5) |
| **PCOS phenotype** | | |
| Hyperandrogenic | 282 (75)^5^ | 288 (76)^4^ |
| Normoandrogenic | 90 (24)^5^ | 86 (23)^4^ |
| **Pregnancy outcomes^*^** | | |
| Late miscarriage | 3 (0.8) | 7 (1.9) |
| Preterm birth | 16 (4.2)^1^ | 30 (7.9) |
| Preeclampsia | 17 (4.5)^2^ | 22 (5.8) |
| Gestational diabetes mellitus | 132 (41)^57^ | 141 (41)^34^ |
| **Mode of delivery^*^** | | |
| Spontaneous vaginal | 263 (70)^2^ | 266 (70) |
| Vacuum extraction | 35 (9.3)^2^ | 37 (9.8) |
| Forceps | 3 (0.8)^2^ | 4 (1.1) |
| Caesarean section | 74 (20)^2^ | 71 (19) |
| **Characteristics at delivery^*^** | | |
| Gestational age (days) | 279 (272-285)^1^ | 280 (272-286) |
| Birth weight (g) | 3560 (3245-3950)^3^ | 3553 (3150-3850)^4^ |
| Birth length (cm) | 50 (49-52)^9^ | 50 (49-52)^11^ |
| Placental weight (g) | 650 (580-766)^41^ | 650 (570-750)^52^ |
| Fetal sex female | 181 (49)^4^ | 184 (49)^3^ |

Continuous variables are reported as median (25th-75th percentile)^m^, and categorical variables as N (%)^m^, where m is the number of missing data points.

**^*^**Comparisons were made by Mann-Whitney U test for continuous variables, and the chi square or Fisher’s exact test for categorical data. There were no significant between-group differences except for preterm birth (P<0.05).

Abbreviations: BMI, body mass index; DBP, diastolic blood pressure; PCOS, polycystic ovary syndrome; SBP, systolic blood pressure.
