# Supplementary material for: Metformin Treatment in PCOS Pregnancies Reduces Maternal Infections and Increases the Risk of Allergies and Eczema in the Offspring: Post Hoc Analyses of Two Randomised Controlled Trials and One Follow‐Up Study
Source: BJOG. 2025 Aug 11;132(12):1823–32. doi: 10.1111/1471-0528.18320 (PMC12501709; doi:10.1111/1471-0528.18320)
Supplement: Supplementary file 6 — Table S3: Characteristics at birth and at 8‐year follow‐up for offspring exposed to metformin or placebo in utero (intention‐to‐treat analysis, PedMet study). [file BJO-132-1823-s004.docx]

**Table S3: Characteristics at birth and at 8-year follow-up for offspring exposed to metformin or placebo in utero (intention-to-treat analysis, PedMet study)**

|  | **Metformin (N=80)** | **Placebo (N=78)** | **P-value** |
| --- | --- | --- | --- |
| **Characteristics at birth** | | | |
| Gestational age (days) | 280 (272-286) | 279 (271-285) | 0.5 |
| Birth weight (g) | 3550 (3260-3930) | 3520 (3100-3910) | 0.6 |
| Birth weight (z-score) | -0.14 (-0.85-0.61) | -0.08 (-0.62-0.67) | 0.5 |
| Birth length (cm) | 50 (49-51)^1^ | 50 (48-52)^1^ | >0.9 |
| Birth length (z-score) | -0.6 (-1.58-0.28)^1^ | -0.33 (-1.18-0.51)^1^ | 0.2 |
| Head circumference (cm) | 36 (35-37)^1^ | 35 (34-36) | **0.02** |
| Head circumference (z-score) | 0.33 (-0.26-0.92)^1^ | 0.19 (-0.65-0.88) | 0.3 |
| Sex female | 40 (50) | 43 (55) | 0.5 |
| **Characteristics at follow-up** | | | |
| Age (years) | 7.9 (6.6-8.7) | 7.8 (6.9-8.8) | 0.6 |
| Weight (kg) | 29 (24-37)^2^ | 28 (24-32) | 0.4 |
| Weight (z-score) | 0.54 (-0.25-1.6)^2^ | 0.21 (-0.57-1.09)^1^ | **0.04** |
| Height (cm) | 128 (122-137)^2^ | 130 (122-136) | >0.9 |
| Height (z-score) | 0.22 (-0.56-0.84)^2^ | 0.03 (-0.64-0.55)^1^ | 0.3 |
| BMI (kg/m^2^) | 17.3 (15.6, 20.3)^2^ | 16.4 (15.2-18.9) | 0.09 |
| BMI (z-score) | 0.52 (-0.27-1.29)^2^ | 0.05 (-0.55-0.87)^1^ | **0.05** |

Continuous variables are reported as median (25th-75th percentile)^m,^ and categorical variables as N (%)^m^, where m is the number of missing data points. Comparisons were made by Mann-Whitney U test for continuous data, and the chi square or Fisher’s exact test for categorical data. Significant P-values are shown in bold.

Abbreviations: BMI, body mass index.
