# Supplementary material for: Metformin Treatment in PCOS Pregnancies Reduces Maternal Infections and Increases the Risk of Allergies and Eczema in the Offspring: Post Hoc Analyses of Two Randomised Controlled Trials and One Follow‐Up Study
Source: BJOG. 2025 Aug 11;132(12):1823–32. doi: 10.1111/1471-0528.18320 (PMC12501709; doi:10.1111/1471-0528.18320)
Supplement: Supplementary file 7 — Table S4: Baseline characteristics and pregnancy outcomes of mothers whose offspring participated in the PedMet study by metformin or placebo randomization (per‐protocol analysis). [file BJO-132-1823-s014.docx]

**Table S4: Baseline characteristics and pregnancy outcomes of mothers whose offspring participated in the PedMet study by metformin or placebo randomization (per-protocol analysis)**

|  | **Metformin (N=74)** | **Placebo (N=71)** | **P-value** |
| --- | --- | --- | --- |
| **Maternal characteristics** | | | |
| Age (years) | 30 (27-32) | 30 (27-34) | 0.5 |
| BMI (kg/m^2^) | 29 (23.7-33.3) | 26.7 (23.4-30.8) | 0.13 |
| Nulliparous | 45 (61) | 40 (56) | 0.6 |
| SBP (mmHg) | 115 (110-128) | 117 (110-124) | 0.6 |
| DBP (mmHg) | 72 (65-80) | 72 (67-79) | 0.9 |
| Smoking | 5 (6.8) | 6 (8.5)^1^ | 0.6 |
| Metformin use at conception | 26 (35) | 20 (28) | 0.4 |
| Asthma | 4 (6.2)^9^ | 1 (1.6)^9^ | 0.4 |
| Allergy | 1 (1.5)^9^ | 1 (1.6)^9^ | >0.9 |
| Eczema | 0 (0)^9^ | 2 (3.2)^9^ | 0.2 |
| **PCOS phenotype** | | | |
| Hyperandrogenic | 54 (73) | 52 (73) | 0.8 |
| Normoandrogenic | 20 (27) | 19 (27) |  |
| **Pregnancy outcomes** |  |  |  |
| Preterm birth | 2 (2.7) | 8 (11) | 0.052 |
| Preeclampsia | 7 (9.5) | 5 (7) | 0.6 |
| Gestational diabetes mellitus | 24 (32)^6^ | 23 (32)^4^ | 0.9 |
| **Mode of delivery** |  |  |  |
| Vaginal delivery | 55 (74) | 42 (59) | 0.11 |
| Vacuum extraction | 7 (9.5) | 10 (14) |  |
| Forceps | 1 (1.4) | 0 (0) |  |
| Caesarean section | 11 (15) | 19 (27) |  |

Continuous variables are reported as median (25th-75th percentile)^m^, and categorical variables as N (%)^m^, where m is the number of missing data points. Comparisons were made by Mann-Whitney U test for continuous data, and the chi square or Fisher’s exact test for categorical data. Significant P-values are shown in bold.

Abbreviations: BMI, body mass index; DBP, diastolic blood pressure; PCOS, polycystic ovary syndrome; SBP, systolic blood pressure.
