# Supplementary material for: Metformin Treatment in PCOS Pregnancies Reduces Maternal Infections and Increases the Risk of Allergies and Eczema in the Offspring: Post Hoc Analyses of Two Randomised Controlled Trials and One Follow‐Up Study
Source: BJOG. 2025 Aug 11;132(12):1823–32. doi: 10.1111/1471-0528.18320 (PMC12501709; doi:10.1111/1471-0528.18320)
Supplement: Supplementary file 9 — Table S6: Maternal and offspring characteristics of participants and non‐participants in the PedMet study. [file BJO-132-1823-s005.docx]

**Table S6: Maternal and offspring characteristics of participants and non-participants in the PedMet study**

|  | **Participants (N=159)** | **Non-participants (N=134)** | **P-value** |
| --- | --- | --- | --- |
| **Maternal characteristics** | | | |
| Age (years) | 30 (27-32) | 29 (25-32) | 0.12 |
| BMI (kg/m^2^) | 28 (24-33) | 28 (24-34) | 0.7 |
| Nulliparous | 93 (58) | 72 (54) | 0.8 |
| SBP (mmHg) | 117 (110-126) | 118 (110-126) | 0.8 |
| DBP (mmHg) | 72 (67-80) | 74 (67-79) | 0.8 |
| Smoking | 11 (7)^1^ | 17 (13) | 0.1 |
| Metformin use at conception | 52 (33) | 40 (30) | 0.6 |
| Asthma | 6 (4.3)^18^ | 5 (4.3)^19^ | >0.9 |
| Allergy | 2 (1.4)^18^ | 1 (0.9)^19^ | >0.9 |
| Eczema | 2 (1.4)^18^ | 1 (0.9)^19^ | >0.9 |
| **PCOS phenotype** | | | |
| Hyperandrogenic | 116 (73) | 106 (79) | 0.4 |
| Normoandrogenic | 43 (27) | 28 (21) |  |
| **Pregnancy outcomes** |  |  |  |
| Preterm birth | 11 (6.9) | 8 (6) | 0.7 |
| Preeclampsia | 12 (7.5) | 7 (5.2) | 0.4 |
| Gestational diabetes mellitus | 51 (35)^12^ | 41 (33)^11^ | 0.8 |
| **Mode of delivery** |  |  |  |
| Vaginal delivery | 110 (69) | 98 (73) | 0.11 |
| Vacuum extraction | 17 (11) | 5 (3.7) |  |
| Forceps | 1 (0.6) | 1 (0.7) |  |
| Caesarean section | 31 (19) | 30 (22) |  |
| **Offspring characteristics at birth** | | | |
| Gestational age (days) | 279 (271-285) | 279 (272-285) | 0.9 |
| Birth weight (g) | 3550 (3190-3930) | 3590 (3260-3990) | 0.3 |
| Birth weight (z-score) | -0.13 (-0.72-0.64) | 0.09 (-0.52-0.72) | 0.3 |
| Birth length (cm) | 50 (49-52)^2^ | 50 (49-51)^2^ | 0.6 |
| Birth length (z-score) | -0.40 (-1.32-0.32)^2^ | -0.53 (-1.04-0.29)^2^ | 0.7 |
| Head circumference (cm) | 36 (34-36.5)^1^ | 35 (34-36.5)^2^ | 0.5 |
| Head circumference (z-score) | 0.28 (-0.43-0.88)^1^ | 0.06 (-0.66-0.88)^2^ | 0.3 |
| Sex female | 84 (53) | 64 (48) | 0.4 |

Continuous variables are reported as median (25th-75th percentile)^m^, and categorical variables as N (%)^m^, where m is the number of missing data points. Comparisons were made by Mann-Whitney U test for continuous data, and the chi square or Fisher’s exact test for categorical data. Significant P-values are shown in bold.

Abbreviations: BMI, body mass index; DBP, diastolic blood pressure; PCOS, polycystic ovary syndrome; SBP, systolic blood pressure.
