# Supplementary material for: Metformin Treatment in PCOS Pregnancies Reduces Maternal Infections and Increases the Risk of Allergies and Eczema in the Offspring: Post Hoc Analyses of Two Randomised Controlled Trials and One Follow‐Up Study
Source: BJOG. 2025 Aug 11;132(12):1823–32. doi: 10.1111/1471-0528.18320 (PMC12501709; doi:10.1111/1471-0528.18320)
Supplement: Supplementary file 10 — Table S7: Incidence of infections during pregnancy, delivery and postpartum in women with PCOS randomised to metformin or placebo, stratified by maternal baseline BMI (per‐protocol analysis, PregMet and PregMet2 studies). [file BJO-132-1823-s009.docx]

**Table S7: Incidence of infections during pregnancy, delivery, and postpartum in women with PCOS randomized to metformin or placebo, stratified by maternal baseline BMI (per-protocol analysis, PregMet and PregMet2 studies)**

|  | *Maternal BMI<25* | | | | *Maternal BMI 25-29.9* | | | | *Maternal BMI≥30* | | | |  |
| --- | --- | --- | --- | --- | --- | --- | --- | --- | --- | --- | --- | --- | --- |
|  | **Metformin**  **(N=99)** | **Placebo**  **(N=127)** | **Odds ratio (95% CI)** | **P-value** | **Metformin**  **(N=82)** | **Placebo**  **(N=100)** | **Odds ratio (95% CI)** | **P-value** | **Metformin**  **(N=135)** | **Placebo (N=105)** | **Odds ratio (95% CI)** | **P-value** | **P-value interaction*** |
| **During pregnancy** | | | | | | | | | | | | | |
| Viral infections | 33 (33) | 45 (35) | 0.91 (0.52-1.58) | 0.7 | 23 (28) | 34 (34) | 0.76 (0.40-1.42) | 0.4 | 38 (28) | 45 (43) | 0.52 (0.30-0.89) | **0.018** | 0.3 |
| Bacterial infections | 18 (18) | 22 (17) | 1.06 (0.53-2.11) | 0.9 | 12 (15) | 23 (23) | 0.57 (0.26-1.22) | 0.2 | 22 (16) | 21 (20) | 0.78 (0.40-1.51) | 0.5 | 0.4 |
| Fungal infections | 3 (3) | 5 (3.9) | 0.76 (0.15-3.19) | 0.7 | 4 (4.9) | 4 (4) | 1.23 (0.28-5.36) | 0.8 | 5 (3.7) | 4 (3.8) | 0.97 (0.25-4.01) | >0.9 | 0.6 |
| Viral, bacterial, and fungal infections | 44 (44) | 65 (51) | 0.76 (0.45, 1.29) | 0.3 | 33 (40) | 50 (50) | 0.67 (0.37-1.21) | 0.2 | 56 (41) | 56 (53) | 0.62 (0.37-1.04) | 0.069 | 0.6 |
| **At delivery or postpartum** | | | | | | | | | | | | | |
| Total infections | 6 (6.1) | 7 (5.5) | 1.11 (0.35-3.44) | 0.9 | 8 (9.8) | 7 (7) | 1.44 (0.49-4.27) | 0.5 | 11 (8.1) | 8 (7.6) | 1.08 (0.42-2.88) | 0.9 | 0.6 |

Categorical variables are reported as N (%). Comparisons were made by logistic regression. Significant P-values are shown in bold. All P-values are nominal without adjustment for multiple testing.

*P-value from logistic regression of the interaction term between maternal baseline BMI and metformin treatment.

Abbreviations: CI, confidence interval; BMI, body mass index; PCOS, polycystic ovary syndrome.
