# Supplementary material for: Metformin Treatment in PCOS Pregnancies Reduces Maternal Infections and Increases the Risk of Allergies and Eczema in the Offspring: Post Hoc Analyses of Two Randomised Controlled Trials and One Follow‐Up Study
Source: BJOG. 2025 Aug 11;132(12):1823–32. doi: 10.1111/1471-0528.18320 (PMC12501709; doi:10.1111/1471-0528.18320)
Supplement: Supplementary file 11 — Table S8: Incidence of infections during pregnancy, delivery and postpartum in women with PCOS randomised to metformin or placebo (intention‐to‐treat analysis, PregMet and PregMet 2 studies). [file BJO-132-1823-s010.docx]

**Table S8: Incidence of infections during pregnancy, delivery, and postpartum in women with PCOS randomized to metformin or placebo (intention-to-treat analysis, PregMet and PregMet 2 studies)**

|  |  |  |  | *Crude analysis* | | *Adjusted analysis** | |
| --- | --- | --- | --- | --- | --- | --- | --- |
|  | **Metformin**  **(N=377)** | **Placebo**  **(N=378)** | **ARD**  **(95% CI)** | **Odds ratio**  **(95% CI)** | **P-value** | **Odds ratio**  **(95% CI)** | **P-value** |
| **During pregnancy** | | | | | | | |
| **Viral infections** | 108 (29) | 133 (35) | -0.07 (-0.13 to 0.001) | 0.74 (0.54-1.00) | 0.054 | 0.74 (0.54-1.00) | 0.052 |
| Respiratory tract infections | 91 (26)^30^ | 120 (33)^15^ |  |  |  |  |  |
| Gastroenteritis | 24 (6.9)^30^ | 27 (7.5)^15^ |  |  |  |  |  |
| Other viral infections^a^ | 5 (1.4)^30^ | 3 (0.8)^15^ |  |  |  |  |  |
| **Bacterial infection** | 61 (16) | 73 (19) | -0.03 (-0.09 to 0.02) | 0.81 (0.55-1.17) | 0.3 | 0.79 (0.54-1.16) | 0.2 |
| Respiratory tract infections | 14 (4)^30^ | 22 (6.1)^15^ |  |  |  |  |  |
| Urinary tract infections | 38 (11)^30^ | 45 (13)^15^ |  |  |  |  |  |
| Other bacterial infections^b^ | 12 (3.5)^30^ | 11 (3.0)^15^ |  |  |  |  |  |
| Use of antibiotics | 59 (17)^30^ | 72 (20)^15^ |  |  |  |  |  |
| **Fungal infection** | 12 (3.2) | 15 (4) | -0.01 (-0.03 to 0.02) | 0.80 (0.36-1.72) | 0.6 | 0.78 (0.35-1.70) | 0.5 |
| Vaginal fungal infections | 10 (2.9)^30^ | 13 (3.6)^15^ |  |  |  |  |  |
| Other fungal infections^c^ | 2 (0.6)^30^ | 2 (0.6)^15^ |  |  |  |  |  |
| **Viral, bacterial, and fungal infections** | 151 (40) | 185 (49) | -0.09 (-0.16 to -0.02) | 0.70 (0.52-0.93) | **0.014** | 0.69 (0.52-0.92) | **0.013** |
| **At delivery or postpartum** | | | | | | | |
| **Total infections** | 30 (8) | 24 (6.3) | 0.02 (-0.02 to 0.05) | 1.28 (0.73-2.24) | 0.4 | 1.26 (0.72-2.21) | 0.4 |
| Fever | 14 (3.7)^2^ | 11 (2.9) |  |  |  |  |  |
| Other infections^d^ | 17 (4.5)^2^ | 13 (3.5) |  |  |  |  |  |

Categorical variables are reported as N (%)^m^, where m is the number of missing data points. Comparisons were made by logistic regression. Significant P-values are shown in bold. All P-values are nominal without adjustment for multiple testing.

^a^Chickenpox (N=1) and herpes simplex (N=4) in the metformin group. Herpes simplex (N=1), herpes zoster (N=1) and viral meningitis (N=1) in the placebo group.

^b^Bacterial vaginosis (N=1), otitis (N=1), conjunctivitis (N=1), skin infection (N=2), dental abscess (N=5) and unspecified infection (N=2) in the metformin group. Bacterial breast infection (N=1), chlamydia (N=1), otitis (N=3), conjunctivitis (N=1), infection in the parotid gland (N=1), pyelonephritis (N=1), tick-borne infection (N=1), dental abscess (N=1) and unspecified infection (N=1) in the placebo group.

^c^Oral candidiasis (N=1) and breast candidiasis (N=1) in the metformin group. Oral candidiasis (N=2) in the placebo group.

^d^Chorioamnionitis (N=6), sepsis (N=1), wound infection after caesarean section (N=4), vulvar wound infection (N=1) and mastitis (N=5) in the metformin group. Chorioamnionitis (N=5), sepsis (N=1), wound infection after caesarean section (N=5) and mastitis (N=2) in the placebo group.

*Adjusted for maternal baseline body mass index.

Abbreviations: ARD, absolute risk differences; CI, confidence interval; PCOS, polycystic ovary syndrome.
