# Supplementary material for: Metformin Treatment in PCOS Pregnancies Reduces Maternal Infections and Increases the Risk of Allergies and Eczema in the Offspring: Post Hoc Analyses of Two Randomised Controlled Trials and One Follow‐Up Study
Source: BJOG. 2025 Aug 11;132(12):1823–32. doi: 10.1111/1471-0528.18320 (PMC12501709; doi:10.1111/1471-0528.18320)
Supplement: Supplementary file 12 — Table S9: Incidence of infections during pregnancy, delivery and postpartum in women with PCOS randomised to metformin or placebo, stratified by maternal baseline BMI (intention‐to‐treat analysis, PregMet and PregMet2 studies). [file BJO-132-1823-s003.docx]

**Table S9: Incidence of infections during pregnancy, delivery, and postpartum in women with PCOS randomized to metformin or placebo, stratified by maternal baseline BMI (intention-to-treat analysis, PregMet and PregMet2 studies)**

|  | *Maternal BMI<25* | | | | *Maternal BMI 25-29.9* | | | | *Maternal BMI≥30* | | | |  |
| --- | --- | --- | --- | --- | --- | --- | --- | --- | --- | --- | --- | --- | --- |
|  | **Metformin**  **(N=125)** | **Placebo**  **(N=144)** | **Odds ratio (95% CI)** | **P-value** | **Metformin**  **(N=98)** | **Placebo**  **(N=111)** | **Odds ratio (95% CI)** | **P-value** | **Metformin**  **(N=154)** | **Placebo (N=123)** | **Odds ratio (95% CI)** | **P-value** | **P-value interaction*** |
| **During pregnancy** | | | | | | | | | | | | | |
| Viral infections | 38 (30) | 46 (32) | 0.93 (0.55-1.56) | 0.8 | 26 (27) | 38 (34) | 0.69 (0.38-1.25) | 0.2 | 44 (29) | 49 (40) | 0.60 (0.36-1.00) | **0.049** | 0.4 |
| Bacterial infections | 22 (18) | 23 (16) | 1.12 (0.59-2.14) | 0.7 | 14 (14) | 25 (23) | 0.57 (0.27-1.16) | 0.13 | 25 (16) | 25 (20) | 0.76 (0.41-1.41) | 0.4 | 0.2 |
| Fungal infections | 3 (2.4) | 5 (3.5) | 0.68 (0.14-2.84) | 0.6 | 4 (4.1) | 5 (4.5) | 0.90 (0.22-3.50) | 0.9 | 5 (3.2) | 5 (4.1) | 0.79 (0.22-2.91) | 0.7 | >0.9 |
| Viral, bacterial, and fungal infections | 50 (40) | 66 (46) | 0.79 (0.48-1.28) | 0.3 | 37 (38) | 55 (50) | 0.62 (0.35-1.07) | 0.087 | 64 (42) | 64 (52) | 0.66 (0.41-1.06) | 0.083 | 0.5 |
| **At delivery or postpartum** | | | | | | | | | | | | | |
| Total infections | 8 (6.4) | 7 (4.9) | 1.34 (0.47-3.92) | 0.6 | 9 (9.2) | 8 (7.2) | 1.30 (0.48-3.60) | 0.6 | 13 (8.4) | 9 (7.3) | 1.17 (0.49-2.92) | 0.7 | 0.5 |

Categorical variables are reported as N (%). Comparisons were made by logistic regression. Significant P-values are shown in bold. All P-values are nominal without adjustment for multiple testing.

*P-value from logistic regression of the interaction term between maternal baseline BMI and metformin treatment.

Abbreviations: CI, confidence interval; BMI, body mass index; PCOS, polycystic ovary syndrome.
