# Supplementary material for: Metformin Treatment in PCOS Pregnancies Reduces Maternal Infections and Increases the Risk of Allergies and Eczema in the Offspring: Post Hoc Analyses of Two Randomised Controlled Trials and One Follow‐Up Study
Source: BJOG. 2025 Aug 11;132(12):1823–32. doi: 10.1111/1471-0528.18320 (PMC12501709; doi:10.1111/1471-0528.18320)
Supplement: Supplementary file 13 — Table S10: Incidence of infections during pregnancy, delivery and postpartum in women with PCOS randomised to metformin or placebo without imputed data (per‐protocol analysis, PregMet and PregMet2 studies). [file BJO-132-1823-s002.docx]

**Table S10: Incidence of infections during pregnancy, delivery, and postpartum in women with PCOS randomized to metformin or placebo without imputed data (per-protocol analysis, PregMet and PregMet2 studies)**

|  |  | |  |  | *Crude analysis* | | *Adjusted analysis** | |
| --- | --- | --- | --- | --- | --- | --- | --- | --- |
|  | **Metformin**  **(N=314)** | **Placebo**  **(N=332)** | | **ARD**  **(95% CI)** | **Odds ratio**  **(95% CI)** | **P-value** | **Odds ratio**  **(95% CI)** | **P-value** |
| **During pregnancy** | | | | | | | | |
| Viral infections | 94 (30) | 124 (37) | | -0.07 (-0.15 to -0.002) | 0.72 (0.52-0.99) | **0.047** | 0.72 (0.51-1.00) | **0.047** |
| Bacterial infections | 52 (17) | 66 (20) | | -0.03 (-0.09 to 0.03) | 0.80 (0.53-1.19) | 0.3 | 0.79 (0.53-1.19) | 0.3 |
| Fungal infections | 12 (3.8) | 13 (3.9) | | -0.001 (-0.03 to 0.03) | 0.98 (0.43-2.18) | >0.9 | 0.97 (0.43-2.19) | >0.9 |
| Viral, bacterial, and fungal infections | 133 (42) | 171 (52) | | -0.09 (-0.17 to -0.02) | 0.69 (0.51-0.94) | **0.02** | 0.70 (0.51-0.95) | **0.023** |
| **At delivery or postpartum** | | | | | | | | |
| Total infections | 25 (8) | 22 (6.6) | | 0.01 (-0.03 to 0.05) | 1.22 (0.67-2.22) | 0.5 | 1.19 (0.65-2.18) | 0.6 |

Categorical variables are reported as N (%). Comparisons were made by logistic regression. Significant P-values are shown in bold. All P-values are nominal without adjustment for multiple testing.

*Adjusted for maternal baseline body mass index.

Abbreviations: ARD, absolute risk differences; CI, confidence interval; PCOS, polycystic ovary syndrome.
