# Supplementary material for: Metformin Treatment in PCOS Pregnancies Reduces Maternal Infections and Increases the Risk of Allergies and Eczema in the Offspring: Post Hoc Analyses of Two Randomised Controlled Trials and One Follow‐Up Study
Source: BJOG. 2025 Aug 11;132(12):1823–32. doi: 10.1111/1471-0528.18320 (PMC12501709; doi:10.1111/1471-0528.18320)
Supplement: Supplementary file 14 — Table S11: Incidence of infections during pregnancy, delivery and postpartum in women with PCOS randomised to metformin or placebo without imputed data (intention‐to‐treat analysis, PregMet and Pregmet2 studies). [file BJO-132-1823-s012.docx]

**Table S11: Incidence of infections during pregnancy, delivery, and postpartum in women with PCOS randomized to metformin or placebo without imputed data (intention-to-treat analysis, PregMet and Pregmet2 studies)**

|  |  |  |  | *Crude analysis* | | *Adjusted analysis** | |
| --- | --- | --- | --- | --- | --- | --- | --- |
|  | **Metformin**  **(N=346)** | **Placebo**  **(N=363)** | **ARD**  **(95% CI)** | **Odds ratio**  **(95% CI)** | **P-value** | **Odds ratio**  **(95% CI)** | **P-value** |
| **During pregnancy** | | | | | | | |
| Viral infections | 108 (31) | 133 (37) | -0.05 (-0.12 to 0.02) | 0.78 (0.57-1.07) | 0.13 | 0.79 (0.57-1.07) | 0.13 |
| Bacterial infections | 61 (18) | 73 (20) | -0.03 (-0.08 to 0.03) | 0.85 (0.58-1.24) | 0.4 | 0.84 (0.57-1.23) | 0.4 |
| Fungal infections | 12 (3.5) | 15 (4.1) | -0.007 (-0.04 to 0.02) | 0.83 (0.38-1.80) | 0.6 | 0.82 (0.37-1.78) | 0.6 |
| Viral, bacterial, and fungal infections | 151 (44) | 185 (51) | -0.07 (-0.15 to 0.001) | 0.75 (0.55-1.00) | 0.051 | 0.74 (0.55-1.00 | **0.05** |
| **At delivery or postpartum** | | | | | | | |
| Total infections | 27 (7.8) | 24 (6.6) | 0.01 (-0.03 to 0.05) | 1.20 (0.68-2.13) | 0.5 | 1.18 (0.66-2.10) | 0.6 |

Categorical variables are reported as N (%). Comparisons were made by logistic regression. Significant P-values are shown in bold. All P-values are nominal without adjustment for multiple testing.

*Adjusted for baseline maternal body mass index.

Abbreviations: ARD, absolute risk differences; CI, confidence interval; PCOS, polycystic ovary syndrome.
