# Supplementary material for: Metformin Treatment in PCOS Pregnancies Reduces Maternal Infections and Increases the Risk of Allergies and Eczema in the Offspring: Post Hoc Analyses of Two Randomised Controlled Trials and One Follow‐Up Study
Source: BJOG. 2025 Aug 11;132(12):1823–32. doi: 10.1111/1471-0528.18320 (PMC12501709; doi:10.1111/1471-0528.18320)
Supplement: Supplementary file 15 — Table S12: Incidence of allergic diseases at 8‐year follow‐up in offspring exposed to metformin or placebo in utero (intention‐to‐treat analysis, PedMet study). [file BJO-132-1823-s015.docx]

**Table S12: Incidence of allergic diseases at 8-year follow-up in offspring exposed to metformin or placebo in utero (intention-to-treat analysis, PedMet study)**

|  |  |  |  | *Crude analysis* | | *Adjusted analysis** | |
| --- | --- | --- | --- | --- | --- | --- | --- |
|  | **Metformin**  **(N=80)** | **Placebo**  **(N=78)** | **ARD**  **(95% CI)** | **Odds ratio**  **(95% CI)** | **P-value** | **Odds ratio**  **(95% CI)** | **P-value** |
| Asthma | 14 (18) | 8 (10) | 0.07 (-0.04 to 0.18) | 1.86 (0.75-4.92) | 0.2 | --- | --- |
| Allergy | 14 (18) | 3 (3.8) | 0.14 (0.04 to 0.23) | 5.30 (1.64-23.7) | **0.011** | 5.37 (1.64-24.2) | **0.011** |
| Eczema | 29 (36) | 15 (19) | 0.17 (0.03 to 0.31) | 2.39 (1.17-5.03) | **0.019** | 2.46 (1.19-5.28) | **0.018** |

Categorical variables are reported as N (%). Comparisons were made by logistic regression. Significant P-values are shown in bold. All P-values are nominal without adjustment for multiple testing.

*Body mass index z-score was included as a covariate to examine whether it mediated the relationship observed in crude analysis.

--- Analysis not performed due to no relationship in crude analysis.

Abbreviations: ARD, absolute risk differences; CI, confidence interval.
